# Semen Quality in Patients with Hematological Malignancies: A Retrospective Monocentric Study in the Perspective of Personalized Oncofertility Medicine

**DOI:** 10.3390/jpm16010011

**Published:** 2025-12-31

**Authors:** Federica Cariati, Maria Grazia Orsi, Anna Capasso, Delia Pagano, Francesca Bagnulo, Gabriele Giuseppe Iorio, Maria Giuseppina Trinchillo, Roberta Ordichelli, Maurizio Guido, Andrea Estrusco, Carlo Alviggi, Alessandro Conforti

**Affiliations:** 1 Department of Public Health, School of Medicine, University of Naples Federico II, 80131 Naples, Italy; mgtrinchillo@gmail.com (M.G.T.); roberta.ordichelli@gmail.com (R.O.); alviggi@unina.it (C.A.); 2 Department of Neuroscience, Reproductive Science and Odontostomatology, University of Naples Federico II, 80131 Naples, Italy; orsimariagrazia.98@gmail.com (M.G.O.); gabrielegiuseppe.iorio@unina.it (G.G.I.); 3 Azienda Ospedaliera Universitaria Federico II, 80131 Naples, Italy; anna.capasso@gmail.com (A.C.); delia.pagano@unina.it (D.P.); francesca.bagnulo@unina.it (F.B.); alessandro.conforti@unina.it (A.C.); 4 Obstetrics and Gynecology Unit, Annunziata Hospital, Via Migliori 1, 87100 Cosenza, Italy; maurizioguido@libero.it; 5 Department of Health Promotion, Mother and Childcare, Internal Medicine and Medical Specialties (PROMISE), University of Palermo, 90127 Palermo, Italy; andrea.etrusco@gmail.com

**Keywords:** human semen, hematological malignancies, male infertility, spermatozoa, semen quality

## Abstract

**Background/Objectives:** The hypothalamic-pituitary-testis axis is known to be dysregulated in patients with hematological malignancies. However, data on the association between the type of hematological malignancies and semen quality are discordant. In the era of personalized medicine, identifying disease-specific patterns of reproductive impairment is crucial to optimize fertility preservation strategies. While patients with leukemia often show a clear deterioration in semen quality, studies on Hodgkin and non-Hodgkin lymphomas have shown that spermatogenesis is not always compromised. Indeed, some patients may present normospermia before treatment. This study aimed to assess semen parameters in males affected by hematological malignancies compared with a non-cancer population and to explore implications for individualized fertility preservation counseling. **Methods:** We performed a retrospective monocentric study including all patients affected by hematological malignancies who underwent fertility preservation at the Maternal and Child Department, Gynecology and Obstetrics, Oncofertility Unit, Federico II of Naples, from January 2017 through December 2024. In total, 247 patients with hematological malignancies and 63 non-cancer males undergoing in vitro fertilization for female tubal factor, selected as a control group, were included in the analysis. Sperm parameters (semen volume, sperm concentration, motility, and morphology) were first compared between the hematological malignancy group and the control group, and then among hematological malignancies classified as Hodgkin lymphoma (HL), non-Hodgkin lymphoma (NHL), and leukemia (L). **Results:** Overall, according to World Health Organization (WHO, 2021) criteria, semen parameters of patients with hematological malignancies were at the 25th percentile, except for motility, which was below the 5th percentile. Significant differences were observed in sperm concentration/mL, total sperm number, and percentage of total sperm motility between the hematological malignancy group and the control group (*p* = 0.0004; *p* = 0.0003; *p* < 0.0001). Based on disease classification, 158 patients had Hodgkin lymphoma, 54 had non-Hodgkin lymphoma, and 35 had leukemia. Significant differences in concentration/mL and total sperm number were found between the Hodgkin lymphoma group and the control group (*p* = 0.003; *p* = 0.001). The percentage of total sperm motility was significantly decreased in all subtypes of hematological malignancies compared with controls, especially in the leukemia group (HL *p* = 0.001; NHL *p* = 0.004; L *p* < 0.001). **Conclusions:** These findings highlight significant impairment of semen quality, particularly motility, reinforcing the role of personalized medicine in tailoring fertility preservation strategies according to malignancy subtype and baseline reproductive risk.

## 1. Introduction

**Hematological Malignancies: Classification and Epidemiology**

Hematological malignancies arise from dysfunctions in normal haematopoiesis and involve the peripheral blood, bone marrow, and lymphatic system [1,2]. These disorders are commonly classified into leukemia, multiple myeloma (MM), non-Hodgkin lymphoma (NHL), and Hodgkin lymphoma (HL) [3]. According to the World Health Organization (WHO), HL is subdivided into classical Hodgkin lymphoma (cHL) and nodular lymphocyte-predominant Hodgkin lymphoma (NLPHL), with cHL further classified into nodular sclerosis, mixed cellularity, lymphocyte-rich, and lymphocyte-depleted subtypes [4,5].

Despite an increase in the absolute number of hematological malignancy cases worldwide between 1990 and 2019, age-standardised mortality rates have declined, reflecting significant advances in prevention, early diagnosis, and treatment [6]. The global incidence of HL increased by 38.66% between 1990 and 2021, although the age-standardised incidence rate remained relatively stable [7]. Recent Global Burden of Disease estimates indicate a rising incidence of Hodgkin lymphoma, particularly in Asian populations, alongside declining global mortality, although increased mortality has been reported in specific countries, including Chile and Ireland [7].

**Risk Factors and Infectious Associations**

Several studies, including analyses of Hodgkin lymphoma following infectious mononucleosis and genome-wide association studies of EBV-defined subgroups, have identified prior Epstein–Barr virus (EBV) infection as a significant risk factor for Hodgkin lymphoma [8,9]. In contrast, childhood exposure to common infectious diseases such as chickenpox, measles, mumps, rubella, or whooping cough appears to be associated with a reduced risk of HL later in life [10].

**Semen Quality in Lymphoproliferative Disorders before Treatment**

In the era of personalized medicine, increasing attention has been directed toward tailoring supportive care strategies according to individual patient characteristics and disease-specific biological features. In this context, oncofertility represents a key field in which personalized risk assessment is essential to guide fertility preservation decisions. The impact of hematological malignancies on semen parameters prior to oncological treatment remains controversial; however, most studies report impaired sperm quality in patients with Hodgkin lymphoma before therapy [11,12], although some investigations have not demonstrated significant alterations [13,14]. Paoli et al. showed that 75% of 519 patients with Hodgkin lymphoma were normospermic prior to treatment. The study also demonstrated that disease stage had a significant impact on semen parameters, with reduced semen volume and lower total sperm count observed in patients with more advanced stages of the disease. Despite these differences, semen parameters in both groups remained within the normal reference ranges established by the World Health Organization (WHO) guidelines [15]. Similarly, Pallotti et al. reported that 82.9% of 222 patients with NHL were normospermic before treatment, although oligozoospermia was significantly more prevalent in aggressive lymphomas than in indolent forms (21.2% vs. 5.1%) [16]. Overall, semen quality appears to vary according to the type and aggressiveness of the hematological malignancy.

**Comparison with Other Malignancies and Effects of Oncological Therapies**

Differences in semen quality have also been observed among various cancer types. Fan et al. reported that Hodgkin lymphoma, non-Hodgkin lymphoma, and leukemia are associated with reduced sperm motility, morphology, and concentration [17]. In contrast, patients with testicular cancer often show lower sperm concentration than those with lymphoproliferative disorders, while maintaining semen parameters within normospermic ranges [14,18]. Given the well-documented gonadotoxic effects of antineoplastic treatments, semen cryopreservation prior to therapy initiation is strongly recommended [19]. Impairment of spermatogenesis may persist at 6 and 12 months following treatment completion, with gradual recovery typically observed after approximately 18 months [16]. However, as demonstrated by Peluso et al., after semen thawing, a reduction in sperm vitality and progressive motility was observed, with a recovery rate of 47.7% and 18.0%, respectively, in patients with hematological cancers [20]. Other studies have also observed a significant reduction in these semen parameters after thawing [21,22].

**Reproductive Outcomes and Study Objectives**

Only a small proportion of patients who cryopreserve semen for oncological indications ultimately use the thawed samples for reproductive purposes. For instance, a recent epidemiological analysis of male cancer patients reported that fewer than 10% of patients who cryopreserved sperm subsequently utilized their samples for assisted reproductive technologies, with approximately half of cycles resulting in a pregnancy [23]. At the In Vitro Fertilization Unit and Gametes Bank of the University Hospital of Bari (Italy), six patients affected by lymphoma thawed their cryopreserved semen for reproductive purposes, achieving an overall pregnancy rate of 33.33%. Similarly, a retrospective analysis conducted at the University Hospital of Nantes observed that, among 82 patients with cancer who used thawed semen following oncological treatment, 30 were affected by hematological malignancies. In this subgroup, pregnancy rates of 30% were achieved using ICSI, while a rate of 18.8% was reported for intrauterine insemination (IUI) [24]. Therefore, the aim of this study was to evaluate semen quality in patients with different hematological malignancies prior to treatment, in order to support a more personalized approach to fertility preservation counseling.

## 2. Materials and Methods

This retrospective, monocentric study designed within a personalized medicine framework, was conducted at the Oncofertility Unit of the Maternal and Child Department, Gynecology and Obstetrics Unit, and Couple Sterility Center of the Federico II University of Naples. A total of 247 male patients diagnosed with hematological malignancies were included. All patients were referred for sperm cryopreservation prior to the initiation of oncological treatment between January 2017 and December 2024. The control group comprised 63 healthy men (mean age: 38.3 ± 5.5 years), partners of couples undergoing in vitro fertilization (IVF) for female-factor tubal infertility. Controls had no personal history of malignancy and had a complete semen analysis available, performed according to the same WHO criteria. Semen parameters were also compared among subgroups of patients diagnosed with Hodgkin lymphoma (n = 158; mean age: 27.7 ± 6.9 years), non-Hodgkin lymphoma (n = 54; mean age: 29.8 ± 8.1 years), and leukemia (n = 35; mean age: 27.8 ± 9.2 years).

Only patients with a histologically confirmed diagnosis of hematological malignancy and a complete pre-treatment semen analysis performed according to the World Health Organization (WHO, 2021) guidelines were included [25]. Exclusion criteria comprised any previous exposure to oncological treatments as well as conditions known to potentially affect sperm quality, including cryptorchidism, clinically significant varicocele, testicular torsion, orchitis, or testicular trauma. Patients with a history of other malignancies or incomplete clinical or seminal data were also excluded.

### 2.1. Semen Analysis

Semen analysis was conducted in accordance with the World Health Organization (WHO) 2021 guidelines [25]. Semen samples were collected by masturbation in a dedicated room adjacent to the andrology laboratory after a recommended period of sexual abstinence of 2–5 days. Immediately after collection, samples were maintained at 37 °C and allowed to liquefy for up to 30 min prior to analysis. Following liquefaction, both macroscopic and microscopic semen parameters were evaluated. Macroscopic assessment included semen volume, appearance, pH, and viscosity. Semen volume was measured using graduated sterile containers, while pH was assessed using calibrated pH indicator strips. Before microscopic evaluation, samples were gently but thoroughly mixed to ensure homogeneity. Sperm concentration was determined using a Makler counting chamber (Sefi-Medical Instruments, Haifa, Israel) under phase-contrast microscopy at ×200 magnification. Total sperm count was calculated by multiplying sperm concentration by ejaculate volume. Sperm motility was assessed manually under phase-contrast microscopy at ×400 magnification using a pre-warmed microscope stage maintained at 37 °C. At least 200 spermatozoa per sample were evaluated and classified as progressively motile, non-progressively motile, or immotile, in accordance with WHO criteria. Sperm morphology was evaluated on air-dried semen smears stained using the Papanicolaou technique. A minimum of 200 spermatozoa per slide were examined under oil immersion at ×1000 magnification, and morphological classification was performed according to strict WHO reference criteria.

For semen cryopreservation, a commercially available cryoprotectant was thawed at room temperature and added drop by drop to the seminal fluid in a 1:1 ratio. Samples were then allowed to equilibrate for 10 min at room temperature and subsequently aliquoted into cryovials previously labeled with patient identification data. The vials were exposed to liquid nitrogen vapor for 30 min to allow controlled cooling and were then directly immersed in liquid nitrogen (−196 °C) for long-term storage.

All semen analyses and cryopreservation procedures were performed by trained embryologists following standardized operating procedures. Laboratory equipment, including phase-contrast microscopes and warming stages, was regularly calibrated and maintained according to manufacturer specifications. Internal quality control measures, including periodic duplicate assessments and inter-observer variability checks, were routinely implemented to ensure consistency and reliability of semen parameter evaluation throughout the study period.

### 2.2. Statistical Analysis

Continuous and categorical data are presented in mean ± standard deviation and percentage, respectively. We used the Shapiro normality test to quantify the distribution of all continuous variables. One-way ANOVA for independent samples was used to assess differences concerning parametric data among groups. Post hoc analysis was performed using Tukey test. Results were analysed using the statistical package SPSS 24 for Windows (Statistical Package for the Social Sciences, IBM, New York, NY, USA). A *p*-value < 0.05 was considered statistically significant.

## 3. Results

Out of 259 patients diagnosed with hematological malignancies, 95% (247/259) were included in the present study and underwent a semen cryopreservation program. According to the WHO Classification of Haematolymphoid Tumours, 64% (158/247) of patients were diagnosed with Hodgkin lymphoma, 21.8% (54/247) with non-Hodgkin lymphoma, and 14.1% (35/247) with leukemia.

The control group consisted of 63 men whose female partners presented with infertility-related factors.

Semen analysis, performed according to the 2021 World Health Organization (WHO) criteria, showed that the semen parameters of patients with hematological malignancies were generally distributed around the 25th percentile, except for total sperm motility, which was below the 5th percentile.

Significantly lower values of sperm concentration (*p* = 0.004), total sperm count (*p* = 0.002), percentage of total sperm motility (*p* < 0.001) and sperm morphology (*p* = 0.043) were recorded in men with hematological malignancies versus controls (Table 1). No statistically significant differences were observed in terms of semen volume.

When analyzing subgroups, patients with Hodgkin lymphoma exhibited a significantly reduced sperm concentration (32.4 ± 32.7 vs. 51.1 ± 38.3; *p* = 0.003) and total sperm number (83.4 ± 91.4 vs. 149.3 ± 156.1; *p* = 0.001) compared to controls.

**Figure 1:**
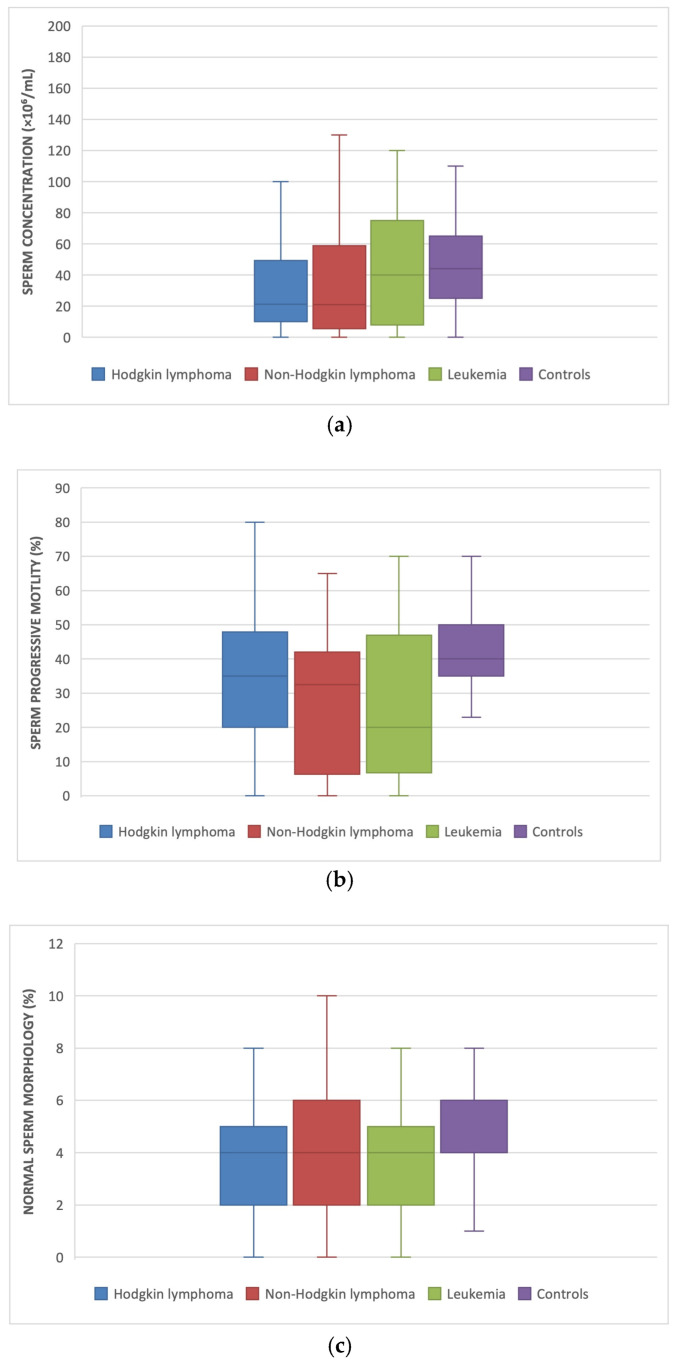
(**a**) sperm concentration, (**b**) total sperm motility and (**c**) normal sperm morphology in diseases and control groups.

In addition, a significant reduction in total sperm motility was observed across all subtypes of hematological malignancies compared to the control group, with the most pronounced decrease in leukemia patients (37.8 ± 23.8 vs. 56.2 ± 14.2; *p* < 0.001), followed by those with Hodgkin lymphoma (47.6 ± 20 vs. 56.2 ± 14.2; *p* = 0.015) and non-Hodgkin lymphoma (46.7 ± 19.1 vs. 56.2 ± 14.2, *p* = 0.041) (Figure 1).

Regarding sperm morphology a trend toward lower percentage of normal sperm was observed in men with leukemia compared with controls (3.9 ± 2.0 vs. 5.1 ± 2.3; *p* = 0.05).

## 4. Discussion

We conducted a comprehensive analysis of semen parameters in patients with hematological malignancies before undergoing oncological treatments, comparing results across the different subgroups as well as with a control group consisting of healthy men whose female partners were affected by infertility factors. Our findings indicate that hematological malignancies exert an early detrimental effect on sperm function, even before the onset of cancer therapy. Affected patients displayed a significant impairment in semen quality compared to healthy controls. This impairment was particularly pronounced in sperm concentration and total sperm count, which were generally distributed around the 25th percentile, whereas total sperm motility was markedly reduced, falling below the 5th percentile.

When examining the individual subgroups, we observed significant differences in sperm concentration and total sperm count between the Hodgkin lymphoma group and the control group only. Additionally, the percentage of total sperm motility was significantly decreased across all subtypes of hematological malignancies relative to controls, with the most pronounced reduction observed in the leukemia subgroup. These findings are consistent with previous studies reporting that patients with Hodgkin lymphoma exhibit lower sperm concentrations compared to fertile populations [17,26,27]. A recent monocentric analysis confirmed compromised semen quality in patients with hematological malignancies compared with non-cancer controls, reinforcing the need for early fertility preservation strategies [28].

Furthermore, Song et al. demonstrated that sperm concentration, total sperm motility, and progressive motility were significantly diminished in cancer patients in comparison with healthy controls. Interestingly, hematological malignancies such as leukemia and lymphoma were associated with higher mean sperm concentrations than those observed in patients with testicular cancer, where the mean sperm concentration was 33.14 ± 24.88 × 10^6^/mL. Although this value falls within the normal range, it is nevertheless significantly lower than that observed in patients with other types of malignancies [28]. A prospective cohort study comparing semen quality and sperm DNA fragmentation index (DFI) in cancer patients before treatment also reported lower sperm concentration and motility in cancer patients relative to controls, reinforcing that cancer itself is associated with impaired semen parameters prior to gonadotoxic exposure [29].

In our cohort, we also observed a significant reduction in sperm motility among patients with leukemia, a finding that aligns with previously published reports [17,28].

Sperm motility represents a highly complex, energy-dependent process that relies on mitochondrial function, axonemal integrity, calcium signaling, and finely regulated redox balance. As detailed by D’Cunha et al., alterations in mitochondrial metabolism and increased reactive oxygen species (ROS) can profoundly impair both total and progressive motility, independently of sperm concentration or morphology. In systemic diseases such as hematological malignancies, chronic inflammation and oxidative stress may therefore directly compromise sperm motility even before the initiation of gonadotoxic therapies, providing a plausible biological explanation for the pronounced motility impairment observed in our leukemia subgroup [30].

A recent retrospective study focused on acute leukemia patients undergoing sperm cryopreservation before treatment found median semen characteristics around the 25th percentile, supporting our observation of early semen impairment in this subgroup [31,32]. These data suggest that even in acute leukemia, where systemic disease effects may be severe, baseline semen quality is often compromised.

The mechanisms by which cancer alters spermatogenesis remain incompletely understood and are likely multifactorial, involving a complex interplay of endocrine and immunological disturbances [33]. In this context, systemic inflammation and the presence of B symptoms such as fever and night sweats—common in patients with lymphoma—have been associated with reduced sperm quality, possibly due to elevated scrotal temperatures and inflammatory cytokine effects on testicular function [32]. A large study of Hodgkin lymphoma patients also highlighted that poor semen quality is strongly associated with the presence of systemic symptoms at diagnosis, underscoring the impact of disease-related inflammation on spermatogenesis [32].

Although alterations in sperm DNA methylation profiles have been predominantly studied post-treatment, emerging evidence indicates that distinct epigenetic signatures are detectable even prior to therapy in patients with Hodgkin disease, suggesting early molecular modifications in spermatozoa that may relate to long-term reproductive health implications [34]. Furthermore, studies on oxidative stress in cancer patients have shown that hematological malignancies can induce high levels of sperm oxidative stress, a factor potentially contributing to impaired motility and sperm function independent of treatment effects (oxidative stress measurement in hematological cancers) [35].

Taken together, these findings support a disease-related impairment of semen quality independent of gonadotoxic treatment. From a personalized medicine perspective, these findings suggest that the impact of hematological malignancies on spermatogenesis is heterogeneous and closely related to disease subtype, systemic inflammatory burden, and underlying biological mechanisms. The observed variability in semen parameters—particularly in sperm motility—supports the need for individualized reproductive risk stratification rather than a uniform oncofertility approach. Integrating semen analysis with clinical and disease-specific characteristics may therefore allow a more tailored assessment of fertility risk prior to oncological treatment.

When interpreting alterations in semen parameters, potential confounding factors must also be considered. Macdonald et al. demonstrated that increasing body mass index (BMI) is associated with reduced sperm concentration and motility, as well as significant alterations in reproductive hormones, even in men without malignancies. These findings highlight the importance of comparing oncological patients with appropriately selected non-cancer controls and suggest that the magnitude of motility impairment observed in our cohort is unlikely to be explained solely by lifestyle or metabolic factors [36]. Data from sperm donor populations further support the interpretation of our findings. Fonseca et al., analyzing semen parameters in a large cohort of sperm donors, reported that sperm motility is among the most sensitive and variable parameters even in men considered representative of optimal reproductive health. The marked reduction in motility observed in patients with hematological malignancies compared with non-cancer controls in the present study therefore suggests a disease-related impairment that exceeds the physiological variability observed in healthy populations [37].

Overall, systemic inflammation, endocrine dysregulation, and oxidative stress appear to act synergistically, contributing to early spermatogenic dysfunction.

In recent years, increasing attention has also been directed toward the role of the seminal microbiome as a mediator between systemic disease, environmental exposures, and sperm quality. Neto et al. highlighted how environmental and systemic factors—including inflammation, oxidative stress, and immune dysregulation—can alter the composition of the seminal microbiome, leading to impaired sperm motility, increased DNA damage, and reduced fertilization potential. In patients with hematological malignancies, disease-related immune activation and systemic inflammatory burden may therefore contribute not only to direct testicular dysfunction but also to microbiome-mediated alterations of the seminal environment, further exacerbating sperm functional impairment even before the initiation of oncological treatment [38].

Emerging evidence on reproductive outcomes in male survivors of hematological malignancies indicates that not only semen quality but also subsequent fertility and live birth rates are importantly affected by both the disease and its treatment. A recent longitudinal follow-up study of 424 men with oncohematological diseases who cryopreserved sperm before therapy reported that among those who expressed a desire for fatherhood, 38% achieved a natural live birth after cancer treatment, and among individuals who used their cryopreserved semen in ART cycles, 51% achieved at least one live birth. Overall, 61% of men interested in fatherhood achieved a live birth either spontaneously or via assisted reproduction (AI/ART) [39]. This underscores the role of sperm cryopreservation not only as a preventive measure but also as a practical means of achieving successful reproductive outcomes in this population.

In this context, advances in assisted reproductive technologies may partially mitigate the negative impact of impaired semen quality in cancer survivors. The introduction of advanced sperm selection techniques—such as physiological ICSI (PICSI), intracytoplasmic morphologically selected sperm injection (IMSI), and microfluidic sperm selection—has been shown to improve the selection of spermatozoa with better motility, morphology, and genomic integrity. These approaches may be particularly relevant for patients with hematological malignancies, in whom baseline sperm impairment and increased oxidative stress are frequently observed, potentially enhancing ART outcomes even when conventional semen parameters are compromised [Advanced Sperm Selection Techniques for Assisted Reproduction] [40].

Follow-up studies in male survivors of hematological malignancies who cryopreserved sperm before undergoing gonadotoxic treatments show that natural conception after therapy is relatively uncommon, whereas assisted reproductive techniques using frozen sperm substantially improve the chances of achieving a live birth [41]. These findings further emphasize the clinical relevance of fertility preservation particularly in patients who may undergo highly gonadotoxic conditioning regimens.

Further supporting these observations, comparative analyses of reproductive outcomes in male cancer survivors undergoing ICSI with cryopreserved sperm have demonstrated live birth rates that are at least comparable to those of non-cancer infertile controls, reflecting the efficacy of ART in this context [42].

Collectively, these data indicate that while hematological malignancies and their treatments significantly affect spermatogenesis and baseline semen quality, appropriately timed fertility preservation coupled with ART provides a meaningful chance of achieving a live birth. Therefore, early counselling and referral for semen cryopreservation at diagnosis remain critical components of comprehensive oncofertility care.

Despite the strengths of this monocentric analysis, including a relatively large cohort and standardized semen assessment performed according to WHO 2021 criteria, several limitations should be acknowledged. First, the retrospective study design inherently limits causal inferences and may be subject to selection bias, as only patients referred for fertility preservation were included. Second, although the control group was carefully selected, it consisted of men from couples undergoing IVF for female-factor infertility, who may not fully represent the general healthy male population. Third, relevant clinical variables potentially influencing semen quality—such as hormonal profiles, systemic inflammatory markers, body mass index and lifestyle factors—were not systematically available and could not be incorporated into the analysis. Additionally, advanced sperm functional parameters, including sperm DNA fragmentation, oxidative stress biomarkers, and epigenetic alterations, were not evaluated, limiting the ability to fully characterize individualized reproductive risk. Finally, reproductive outcomes were not directly assessed in this cohort, precluding correlations between baseline semen impairment and long-term fertility success. These limitations highlight the need for prospective, integrated studies combining clinical, biological, and molecular data to further refine personalized oncofertility strategies.

## 5. Conclusions

In conclusion, hematological malignancies are associated with significant impairment of semen quality prior to oncological treatment, with sperm motility being the most affected parameter. These findings indicate that fertility risk is already present at diagnosis and varies according to disease characteristics, supporting the need for individualized reproductive risk assessment within oncofertility care.

### 5.1. Future Directions

Future prospective, multicentric studies with larger and more balanced cohorts are warranted to confirm these findings and enhance external validity. Longitudinal investigations evaluating semen quality before, during, and after oncological treatments are needed to better characterize spermatogenic damage and recovery. The integration of endocrine, inflammatory, and molecular sperm biomarkers may further clarify disease-specific mechanisms and improve personalized fertility preservation strategies.

### 5.2. Clinical Implications

The present findings have relevant clinical implications for the management of reproductive health in male patients with hematological malignancies. The demonstration of compromised semen quality prior to treatment, particularly in terms of sperm motility, underscores the importance of early fertility counseling and prompt referral to oncofertility services at diagnosis. Integrating semen analysis into personalized oncofertility pathways may allow clinicians to tailor fertility preservation strategies according to disease subtype and individual reproductive risk, supporting the routine incorporation of fertility preservation into standard hematological oncology care through a multidisciplinary approach.

## Figures and Tables

**Table 1:** Semen parameters between hematological cancer and control groups.

	5° Percentile (WHO 2021)	Hodgkin Lymphoma N = 158	Non-Hodgkin Lymphoma N = 54	Leukemia N = 35	Control N = 63	*p*-Value
Volume (mL)	1.4	2.8 ± 1.6	2.9 ± 1.6	2.9 ± 2.1	2.8 ± 1.2	0.96
Sperm concentration ×10^6^/mL	16	32.4 ± 32.7	36.9 ± 38.6	45.1 ± 41.9	51.1 ± 38.3	0.004
Total sperm motility (%)	42	47.6 ± 20	46.7 ± 19.1	37.8 ± 23.8	56.2 ± 14.2	<0.001
Progressive motility (%)	30	33.8 ± 17.9	33 ± 17.8	26.5 ± 21.5	43.7 ± 14.2	<0.001
Normal morphology (%)	4	4.1 ± 2.6	4.7 ± 2.8	3.9 ± 2.0	5.1 ± 2.3	0.043

## Data Availability

The data presented in this study are available on reasonable request from the corresponding author. The data are not publicly available due to privacy and ethical restrictions.

## Abbreviations

The following abbreviations are used in this manuscript:

HL	Hodgkin lymphoma
NHL	non-Hodgkin lymphoma
L	leukemia
MM	multiple myeloma
cHL	classical Hodgkin lymphoma
NLPHL	nodular lymphocyte-predominant Hodgkin lymphoma
EBV	Epstein-Barr virus
IVF	In vitro fertilization
ICSI	Intracytoplasmic Sperm Injection
IUI	Intrauterine insemination
WHO	World Health Organization
